# Continuous, self-sustaining smouldering destruction of simulated faeces

**DOI:** 10.1016/j.fuel.2016.11.014

**Published:** 2017-02-15

**Authors:** Ivo Fabris, Daniel Cormier, Jason I. Gerhard, Tomek Bartczak, Mark Kortschot, Jose L. Torero, Yu-Ling Cheng

**Affiliations:** a Department of Civil and Environmental Engineering, University of Western Ontario, London, Ontario N6A 5B9, Canada; b Centre for Global Engineering and Department of Chemical Engineering and Applied Chemistry, University of Toronto, Toronto, Ontario M5S 3E5, Canada; c School of Civil Engineering, The University of Queensland, Brisbane 4072, Australia

**Keywords:** Smouldering combustion, Faeces, Remediation, Waste management

## Abstract

A new approach for the rapid destruction of human waste using smouldering combustion is presented. Recently, self-sustaining smouldering combustion was shown to destroy the organic component of simulated human solid waste and dog faeces resulting in the sanitization of all pathogens using a batch process (Yermán et al., 2015). Here, a continuous smouldering process is demonstrated for the first time, allowing for a much smaller reactor size and much less energy input per mass of waste treated. The self-sustained smouldering of simulated human faeces mixed with sand is evaluated over long periods (more than 16 h) based on a single ignition. The key process of intermittent self-sustained smouldering, in which the reaction is terminated and restarted by only turning the air off and on, is demonstrated. Experiments examine the influence of two key operator controls: airflow rate and set elevation of the quasi-steady-state smouldering front in a 37 cm high reactor. Quasi-steady-state fuel destruction rates from 93 g/h to 12 g/h were achieved by varying the superficial flow velocity from 7.4 cm/s to 0.11 cm/s, the latter with a velocity approximately an order of magnitude lower than possible for a self-sustaining reaction in an equivalent batch system. Excess energy of up to 140 J/g of sand was recovered from the clean sand produced in each cycle, which could be used to further increase the energy efficiency of this novel waste treatment system.

## 1 Introduction

A technology is needed that is capable of the rapid and inexpensive destruction of human excreta as a sanitary solution for developing countries [2]. This is due to the adverse effects that human waste has on public health [2], [3]. Current treatment methods such as composting and pit latrines are problematic: their long term storage of the waste, combined with poor construction and lack of maintenance results in the leakage of pathogens that contaminate ground and surface waters [3]. The expensive sewage infrastructure and treatment plants common in industrialized countries are infeasible for developing countries.

Incineration and pyrolysis units have been proposed as possible solutions. However, the high moisture content of human faeces, which varies between 65% [4] and 85% [5] wet-basis for a healthy person, reduces its net energy content and therefore requires drying. For instance, the energy content of a faeces sample was measured to be 5.9 kJ/g wet (75.6% moisture content) and 24.3 kJ/g dry [6]. A small scale incinerator tested by Niwagaba et al. [7] concluded that faeces had to be dried to below 10% moisture content and use supplemental fuel for optimal operation. In a pyrolysis unit, the feaces needs to be heated to between 300 °C and 750 °C [8]. Pyrolysis is an endothermic reaction, requiring input energy to drive the reaction, in addition to the energy consumed in drying and heating the fuel. Both incineration and pyrolysis are energy intensive solutions, which increases their operational cost.

Smouldering combustion has the potential to be a low energy, low cost, effective method to destroy faeces. Smouldering is a heterogenous oxidation reaction that takes place on the surface of the fuel. The reaction is limited by the rate of oxygen that can diffuse into the fuel surface [9], [10] resulting in low temperatures and slow reaction rates relative to flaming combustion. Smouldering is characterized as self-sustaining when the oxidation reaction generates enough energy, in the form of heat, to overcome heat losses and sustain the propagation of the reaction indefinitely [11].

Smouldering typically occurs in porous materials such as charcoal, peat [12], and polyurethane foam [13]. Organic solids and liquids that exhibit minimal permeability to air will not smoulder. However, they can be susceptible to smouldering when commingled with an inert porous material to create a permeable mixture [14]. This was demonstrated in a bench-top batch reactor for coal tar [15], industrial organic liquid contaminants present in soil [14], [16] and most recently, for bio-solids waste from wastewater treatment plants [17].

**Fig. 1:**
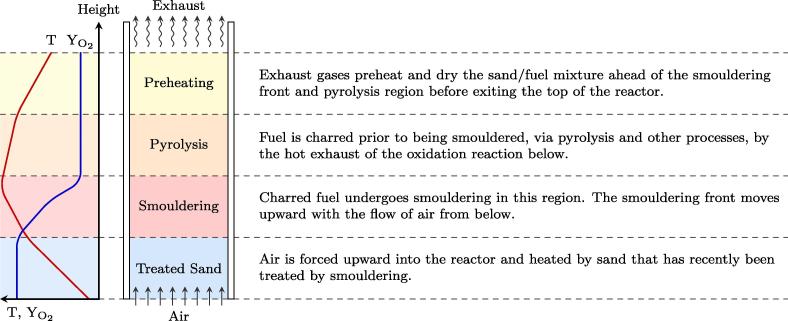
Illustration of the different processes occurring in the smouldering reactor at steady-state shown alongside a simplified plot of the corresponding temperature (T) and gas stream oxygen concentration (${\text{Y}}_{{\text{O}}_{2}}$) versus reactor height. The temperature profile is of a continuous process and differs from a batch reaction (shown in [20]). The regions are not to scale.

A simplified illustration of the smouldering processes is presented in Fig. 1. The temperature (T) and oxygen profiles (${\text{Y}}_{{\text{O}}_{2}}$) are plotted alongside a depiction of the corresponding regions within the reactor: treated sand zone, smouldering zone, pyrolysis zone, and preheating zone. The smouldering zone consist primarily of smouldering char while the pyrolysis zone corresponds to a complex series of primarily endothermic reactions ahead of the smouldering front. The effective heat transfer between the smouldering reaction and fuel allows for higher quenching limits (i.e., less energy content, higher moisture content) than possible for flaming combustion [18], [19].

Forward smouldering as a means to treat human faeces was demonstrated in a batch reactor by Yermán et al. [1]. In that work, smouldering of faeces mixed with sand was shown to destroy all the organic component of the mixture, both with simulated human faeces and dog excreta, while sanitizing all pathogens via long residence times (>20 min) at high temperatures (>400 °C) [1]. The study found self-sustained smouldering can be achieved in a batch reactor for Darcy fluxes (i.e., volume of air per cross sectional area of reactor per time) between 0.6 cm/s and 6.5 cm/s and sand-to-fuel ratios between 2.75:1 g/g and 11.9:1 g/g wet basis. The maximum moisture content that can be smouldered in a batch process was found to be dependent on the fuel pack height: 60–70% for a pack height of 98 cm and 75% for a pack height of 30 cm. Energy production from the exothermic oxidation reaction using high moisture content fuel is one of the main advantages of this process. The recovery of energy from condensable emissions may also be able to enhance the efficiency of this technology [20].

All previous work has used intentional smouldering treatment as a batch process. In the context of designing a toilet in accordance with the Reinvent the Toilet Challenge [2], a continuous process would have numerous benefits. For example, external energy for ignition would be required much less frequently. Also, ther reactor could be much smaller, since storing waste for periodic batch treatment is unnecessary. Furthermore, the ability to continuously vary the destruction rate would allow a continuous system to adjust to variability in usage and loading rates.

The goal of this work is to demonstrate and quantify the performance of the first continuous, self-sustaining smouldering process. The objectives to achieve this goal include (1) demonstrating “intermittent self-sustained smouldering”, in which the reaction is terminated and reignited without adding energy, (2) quantifying the metrics of a continuous smouldering reaction and comparing those to a batch system, and (3) quantifying the recoverable heat from the discharged, treated sand. The scale of the experiments in this work at are at the full scale of intended application: a single family toilet. Overall, this work lays the foundation for continuous, low energy solid waste treatment.

## 2 Experimental method

### 2.1 Apparatus

The apparatus has a similar layout as the batch reactor used in [1] but is modified to allow the extraction of sand from the bottom. The smouldering reaction occurs in a cylindrical 304 stainless steel reactor 37.2 cm tall with an inner diameter of 6.25 cm and a wall thickness of 0.05 cm. The length of the reactor was insulated with alumina-silicate fiber insulation (Fiberfrax manufactured by Unifrax) housed in a stainless steel jacket with an inner diameter of 16.5 cm. A plug heater, consisting of two 200 W Zesta cartridge heaters (0.64 cm diameter × 5.6 cm length), is inserted 3.5 cm above the bottom of the sand bed. This is the full scale system, designed for the treatment of the waste from a single family.

The reactor is instrumented with nine thermocouples, evenly spaced 2.54 cm apart vertically, starting 6.0 cm above the bottom of the sand bed. The thermocouples (Omega K-type KMQIN-125U-6) are inserted into the reactor horizontally approximately 7 ± 2 mm from the reactor wall. It is acknowledged that most studies have used centerline thermocouples in batch reactors and a comparison between centerline and near-wall temperatures during smouldering is included. The temperature is recorded with an Agilent Technologies 34980A Multifunction Switch-Measure Unit sampling every 2 s. The rate of airflow pushed through the reactor is set using a mass flow controller (Omega FMA5423) with a range of 0–15 standard liters per minute (SLPM).

The carbon monoxide, carbon dioxide, and oxygen concentration of the emissions were measured for select experiments using an ADC MGA3000 Gas Analyzer. The gas was measured in real-time during the experiment and the gas is sampled centerline via a tube inserted at the top of the reactor.

### 2.2 The fuel

The composition of the fuel used, detailed in Table 1, is based on the recipe developed by Wignarajah et al. [4] for NASA to simulate human faeces. The recipe simulates the mechanical properties, water retention capacity, and energy content of human faeces. The energy content of the fuel, based on bomb calorimetry measurements for each ingredient of the surrogate faeces, is 20.6 kJ/g dry; this lies within the 17.6–25.1 kJ/g dry range typical of human faeces [4], [6], [21].

The fuel is mixed in batches equivalent to 200 g of dry mass with the moisture content of the fuel controlled by varying the mass of water to be added. The fuel is mixed (KitchenAid KSM7581WH) until homogenous. The silica sand (mean grain size = 1.18 mm, well sorted, Hutcheson) is added slowly to the mixing bowl until the sand/fuel mixture is homogeneous. The mass of sand added is dictated by the desired sand-to-fuel ratio. The fuel moisture content and sand-to-fuel ratio are not varied for these experiments: 33% wet-basis and 24:1 g/g dry mass, respectively. These conditions are selected because they have been demonstrated to result in robust smouldering in a batch reactor [1], offering consistent smouldering conditions as a baseline for study. Robust in this context means that the reaction is not close to quenching, which means that the energy generated, stored, and recycled at the front considerably exceeds the energy lost through the walls and exhaust of the reactor. Furthermore, large sand-to-fuel ratio dilutes the fuel, permitting (i) a low mass destruction rate, which helps maintain a reaction even during low load periods, and (ii) increases the potential for heat storage and thus heat recovery from the sand.

### 2.3 Experimental procedure

The bottom of the reactor is filled with clean sand until a thin layer of sand just covers the heater plugs. The sand/fuel mixture is then loaded into the reactor to approximately 12.7 cm above the initial “trigger” thermocouple (defined below).

The heater was set to 200 W to start the ignition process. The bottom of the reactor is heated until the first thermocouple (TC1), 2.5 cm above the heater and 6.0 cm from the bottom, reaches 250 °C and the airflow is then initiated, delivered from a compressor attached to a mass flow controller. Ignition is verified by a sharp increase in temperature at TC1, and the heater is turned off once the temperature at TC1 has peaked. This initiates the smouldering reaction. This ignition process only occurs once at the outset of each experiment.

**Fig. 2:**
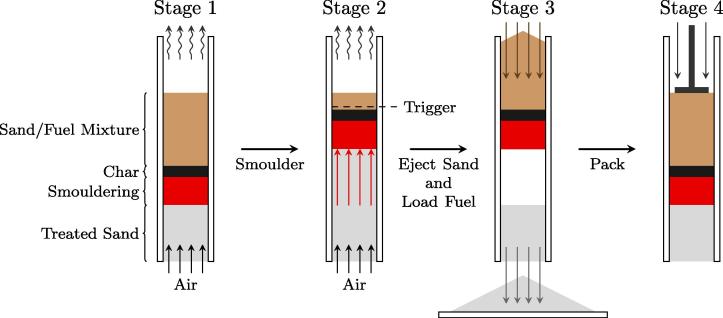
An illustration of continuous reactor operation divided into four stages. Stage 1: the air is turned on and smouldering commences. Stage 2: the smouldering/char front has propagated upwards until the temperature at the trigger height reached 250 °C. Stage 3: the airflow is turned off terminating the forward propagation of the smouldering reaction; clean sand is removed and fuel is added at the top of the reactor. Stage 4: The char layer and virgin fuel/sand mixture is pushed down.

Thereafter, continuous smouldering is achieved by operating successive cycles of the reactor. One cycle comprises two-phases of operation: (i) upwards (forward) smouldering propagation, followed by (ii) downwards sand conveyance. The detailed operation of a cycle, depicted in four stages, are illustrated in Fig. 2. The steps that make up a cycle are:

Described so far, the process is identical to that for smouldering as a batch process. Here, however, continuous smouldering is achieved by operating the reactor in successive “cycles”. One cycle comprises two-phases of operation: (i) upwards (forward) smouldering propagation, followed by (ii) downwards sand conveyance. The detailed operation of a cycle, depicted in four stages, is illustrated in Fig. 2. The steps that make up a cycle are:

Stage 1: **Air initiation**
  Step 1: Air is turned on, igniting (first cycle) or reigniting (each subsequent cycle) the smouldering reaction.
Stage 2: **Forward propagation**
  Step 2: The reaction propagates upward until the selected “trigger height” is reached. The trigger height is the height in the reactor chosen which dictates when to terminate upward propagation. This height corresponds to a chosen thermocouple and the trigger occurs when the selected temperature is reached, 250 °C for these experiments. Note that this is in the preheating zone ahead of the smouldering reaction; the smouldering front is typically approximately 5 cm lower than the trigger height.
Stage 3: **Clean sand discharge**
  Step 3: Air is turned off, rapidly arresting the forward propagation of the smouldering reaction. This is done to permit clean sand to be extracted.
  Step 4: The sand exit cap at the bottom of the reactor is removed. Approximately 170 g of clean sand, equivalent to 3.8 cm of height for this reactor, is discharged from the bottom of the reactor.
  Step 5: Enough new sand/fuel mixture is added to the system to reestablish a 12.7 cm pack height above the trigger thermocouple.
Stage 4: **Downward reposition of char and virgin fuel**
  Step 6: The added sand/fuel mixture is pushed down manually with a 5.0 cm diameter metal plunger. This is necessary because a char layer (the pyrolyzed fuel that smoulders) forms which sticks to the reactor walls and prevents the mixture from flowing downward strictly by gravity. In an automated system this step would be mechanized, however manual implementation was most straightforward for this initial study.
  Step 7: Repeat **Step 1**.

Reactor temperatures typically increase with subsequent cycles until a quasi-steady-state is reached. Quasi-steady-state is here defined as when the peak temperatures of all the thermocouples are unchanging over multiple, sequential cycles. In particular, the temperature closest to the bottom, TC1 at 6 cm, is used to determine quasi-steady-state because it is the last thermocouple to reach a constant average peak temperature over multiple cycles.

Recall that one objective of the study is to quantify the energy extracted in the treated sand. This was achieved by having the extracted sand enter a water bath. The bath temperature was measured before and after receiving the sand, thereby allowing the average recoverable sand energy to be calculated using the heat capacity of the water.

The recoverable sand energy was evaluated as a function of the trigger height. This is important since trigger height is a design variable and it is useful to maximize the recovered energy. After a quasi-steady-state had been established at the current trigger height, the smouldering front was lowered further one thermocouple height by removing 268 g of sand (6.4 cm height drop) at Step 4. The reaction then propagated upwards until the new trigger thermocouple reached 250 °C and normal operation resumed. Note that trigger heights were only ever decreased; thus the system was always shifted from more robust smouldering conditions towards those that were less robust.

### 2.4 Experimental plan

The airflow rates, expressed as the superficial flow velocity or Darcy’s flux, were chosen to be 1.85 cm/s, 3.7 cm/s, and 7.4 cm/s. All experiments and the corresponding trigger heights studied in each experiment are listed in Table 2. There is overlap in experiments E1, E2, and E3 for trigger heights to test the variability between experiments. Note that a typical cycle at 7.4 cm/s airflow took on average 4.5 min while at 1.85 cm/s took 17.5 min. Thus a typical experiment could accomplish approximately 111 cycles at high airflow but only 34 cycles at low airflow. Since it required between 9 and 16 cycles to achieve quasi-steady-state, this meant that low airflow rate experiments could test less trigger heights than high airflow rates, as reflected in Table 2.

## 3 Results and discussion

### 3.1 Temperature histories

**Fig. 3:**
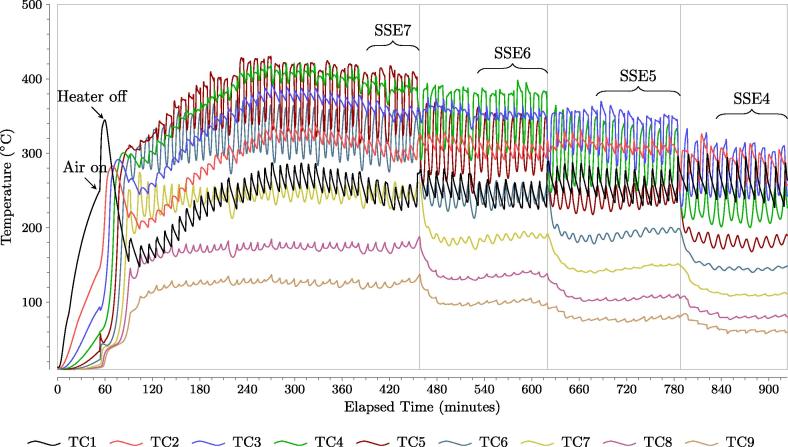
Temperature histories of Experiment E3 for different thermocouple heights. The airflow rate used is 3.7 cm/s. The trigger thermocouples tested are: TC7, TC6, TC5, and TC4, equivalent to 21.2 cm, 18.7 cm, 16.2 cm, and 13.6 cm from the bottom of the reactor, respectively. The quasi-steady-state energy (SSE) portion of the test for each trigger thermocouple is labeled as SSE7, SSE6, SSE5, and SSE4.

The temperature histories for experiment E3 are presented in Fig. 3. The bottom pack of the fuel is heated until the first thermocouple (TC1 at 6 cm) reaches 250 °C which occurred 53 min into the test. The reactor is then sparged with air to ignite the smouldering reaction, resulting in a sharp increase in temperature in TC1 at 53 min (labeled as “Air on” in Fig. 3). Once TC1 has peaked, labeled as “Heater off” in Fig. 3, the heater power is turned off. The reaction is then allowed to propagate upwards in the reactor, characterized by consecutive, overlapping temperature peaks typical of a batch process [1], [14], [15], [16]. This occurs until the temperature at the initial trigger height of 21.2 cm (TC7) reaches 250 °C, which occurs at 90 min and at which point continuous operation commences.

Each dip and then rise in temperature in Fig. 3 represents one process cycle. The initial decrease in temperatures at all the thermocouples simultaneously is the system response to the air flow being turned off in Stage 2 of the cycle (illustrated in Fig. 2). As has been shown previously [14], eliminating the input of oxygen results in the effective cessation of oxidation reactions and constant heat loss. The second temperature decrease results from the downward movement of relatively fresh sand/fuel in Stage 4 of the cycle. The subsequent rapid rise in temperature reflects the systems’s response to the restarting of air flow in Stage 1 of the subsequent cycle. Reignition of the smouldering reaction is expected as long as the peak temperature within the reaction zone (red region in Fig. 2) has not fallen below the ignition temperature.

The reactor peak temperatures are observed to increase after ignition until the reaction reaches a plateau at approximately 300 min. This is not observed in batch smouldering experiments and represents the quasi-steady-state of the reactor materials, and the heat losses of the system, with the reaction. Quasi-steady-state operation, defined as statistically constant peak temperature at TC1 as is the last thermocouple peak to stabilize, is achieved at 383 min. Quasi-steady-state is maintained, using 21.2 cm (TC7) as the trigger height, for 32 cycles until 444 min; this steady-state period is labeled SSE7 in Fig. 3.

The front was then moved to a lower trigger height, 18.7 cm (TC6) and the reactor reached a new quasi-steady-state at 523 min. The peak temperatures were observed to decrease as the front is lowered, as shown in Fig. 3. This is further discussed below. The front lowering procedure was repeated for trigger heights of 16.2 cm (TC5) and 13.6 cm (TC4) for this experiment. As expected, the time to reach steady-state is longest after ignition (41 cycles), while subsequent changes in reactor operation are observed to require only 11 cycles on average to re-establish a new steady-state. This is attributed to the fact that the initial energy stored in the thermal mass of the reactor is large relative to the energy difference between steady-states.

### 3.2 Quasi-steady-state smouldering

**Fig. 4:**
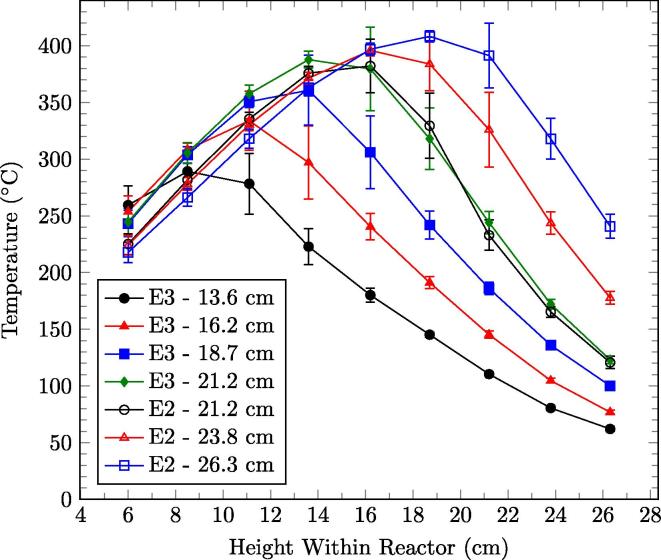
Reactor temperature profiles of experiments E2 and E3 for varying smouldering front location (imposed by the tested trigger height), averaged over the quasi-steady-state period, as a function of reactor height from the bottom of the reactor. The error bars represent one standard deviation in each direction from the averaged samples. These experiments all use a common fuel moisture content, sand-to-fuel ratio and superficial airflow velocity (3.7 cm/s).

The average quasi-steady-state temperature profile along the reactor height for varying smouldering front location, fixed by their trigger heights, is shown in Fig. 4. The two tests presented have identical parameters for superficial airflow velocity, fuel moisture content, and sand-to-fuel ratio. Experiments E2 and E3 have 21.2 cm (TC7) as an overlapping smouldering front location, which demonstrates repeatability between these experiments. The peak reactor temperature decreases with smouldering front height above the bottom of the reactor; the lowest observed peak temperatures occur when the smouldering front is near the base of the reactor with the smouldering front approximately 8 cm (trigger height of 13.6 cm or TC4).

**Fig. 5:**
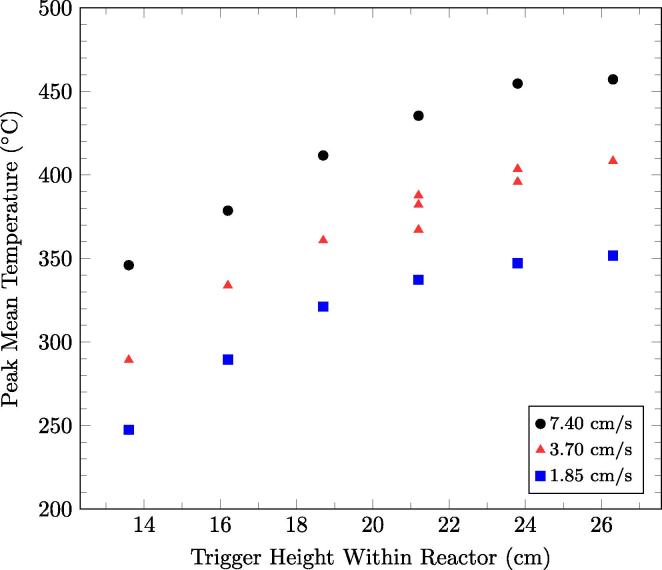
The near-wall peak mean temperatures of the temperature profiles as a function of smouldering front location, fixed by the trigger height, above the bottom of the reactor for different superficial airflow velocities.

The peak mean temperature as a function of smouldering front location for the superficial airflow velocities tested is plotted in Fig. 5. This quantifies the extent to which the peak mean temperature decreases with smouldering front location: about 100 °C for a 10 cm reduction in the quasi-steady-state location of the front. Recall that the smouldering front location is typically 5 cm below the trigger thermocouple height. The trend also shows a non-linear relationship in which the temperature decrease is more severe as the front approaches the bottom of the reactor. The decrease in peak temperature as a function of smouldering front location is attributed to the removal of heat in the treated sand available to preheat the incoming air, thus reducing the reactant temperatures and reaction rates. This suggests that the extracted sand energy has more impact when the front is lower in the reactor, which is further explored in the next section. The figure further demonstrates that peak temperature is a function of the injected air flux; this matches expectations from batch studies [1], [16]. The implications for continuous smouldering performance is explored in Section 3.4.

**Fig. 6:**
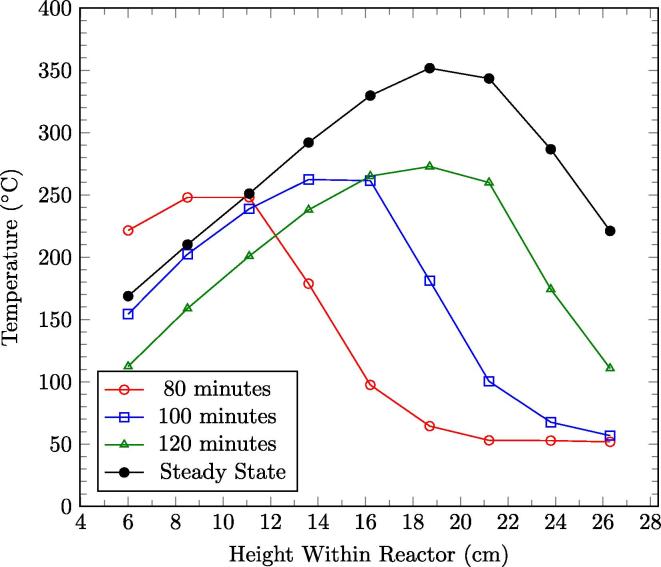
The temperature profiles as a function reactor height of different times between ignition and the first cycle. The average quasi-steady-state temperatures, using 26.3 cm as trigger height, are plotted for comparison.

Previous smouldering studies conducted with this technology used a batch processes in a bench-top reactor [1], [14], [15], [16]. This is equivalent to the forward smouldering phase of the first cycle in these experiments. Fig. 6 presents the temperature profiles for experiment E6 as the reaction propagates upward in the reactor before cyclical operation commences. The profiles for 80 min, 100 min, and 120 min are typical of a batch process: they demonstrate a relatively narrow preheating zone and sharp reaction front propagating up the column. This is typical of a reaction leading smouldering wave [22]. The relatively constant temperature plateau behind the front represents oxidative fuel consumption and the temperature decline towards the column base represents convective cooling. These three profiles correspond to temperature profiles from recent smouldering batch studies [1], [14], [16], [15].

The temperature profile of quasi-steady-state smouldering during continuous operations, also shown in Fig. 6, reveals a different shape. This is the first temperature profile published of average behavior over many cycles in a smouldering reactor. It reveals that the average shape is more symmetric; this is attributed to the localized smouldering front in the reactor that allows the heat to propagate into the virgin fuel over multiple cycles. In addition, the mean peak temperatures are higher for quasi-steady-state, with maximum temperatures of 351 °C and 273 °C for continuous and batch (i.e., first half cycle) respectively for this test. This is attributed to the build up of heat in the reactor that results in higher smouldering velocities and consequently higher heating rates.

**Fig. 7:**
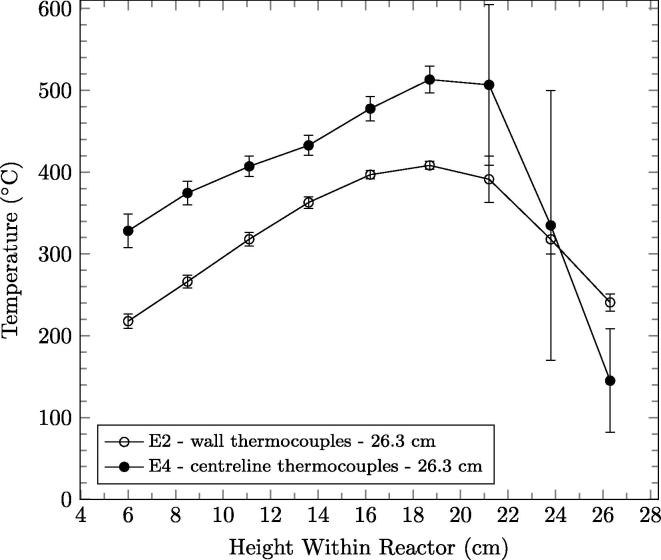
Temperature profiles, averaged of the quasi-steady-state period with error bars representing one standard deviation, measured at the centerline and wall of reactor for trigger heights of 26.3 cm.

The temperature measurements are taken near to the reactor wall (7 ± 2 mm). There is a radial temperature gradient and thus, the temperatures reported are lower than those at the center of the reactor. Fig. 7 shows the centerline versus wall temperature profiles for the same trigger height (26.3 cm). The mean peak temperature of the centerline measurements are approximately 500 °C which is consistent with batch studies using this fuel [1], [20]. The centerline measurements are approximately 105 °C higher than near-wall temperature measurements. However, larger differences than this (e.g., up to 195 °C) are observed at the peak when the front is near the bottom of the reactor and very little difference is observed at the leading edge of the preheating zone. The difference in temperature is attributed to heat losses to the environment via the reactor wall. Because the peak temperatures measured at the centerline and near-wall temperature are well-correlated, near-wall temperatures are a valid approach to tracking the location of the front.

### 3.3 Ejected sand energy

**Fig. 8:**
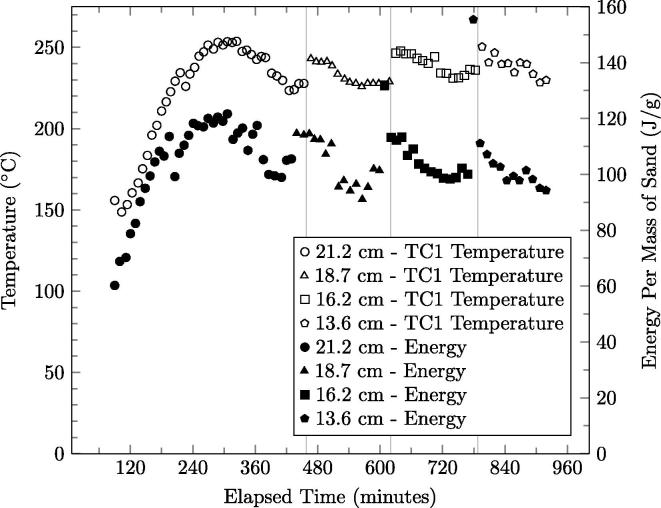
The energy per gram of extracted sand and the temperature measured 6 cm from the bottom of the reactor (TC1) for Experiment E3 with a superficial airflow velocity of 3.7 cm/s.

The average recoverable energy of the ejected sand and the corresponding temperature measured 6 cm from the bottom of the reactor (TC1) as a function of test time is presented in Fig. 8. The energy increases until 382.9 min, consistent with the reactor reaching quasi-steady-state as also observed in Fig. 3. The temperature at TC1 follows the same trend, demonstrating correlation with extracted energy, as expected. The quasi-steady-state temperature profiles for E3 are identified in accordance to the quasi-steady-state energy, labeled SSE (TC number) in Fig. 3.

When the smouldering front is lowered toward a new trigger thermocouple, the energy measurement immediately following increases sharply; this is attributed to a higher average energy in the larger portion of sand removed from the reactor, 285 g as opposed to 170 g, due to the sand’s closer average proximity to the smouldering front. The ejected sand energy then stabilizes after typically 5–8 cycles, indicating that the system has reached a new quasi-steady-state.

**Fig. 9:**
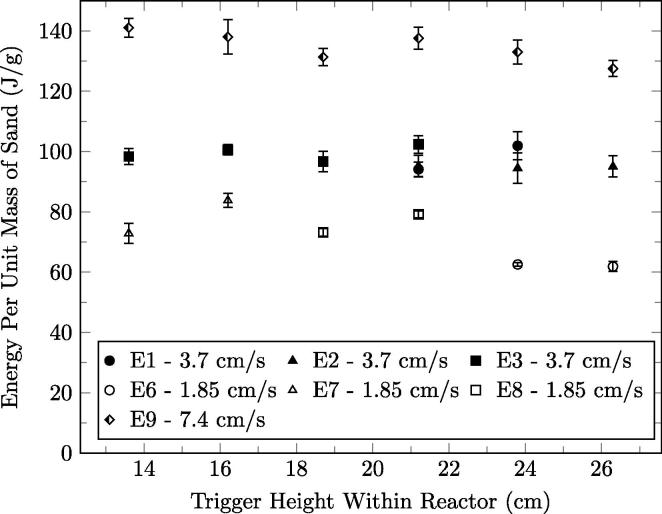
The quasi-steady-state energy per gram of ejected sand for 7.4 cm/s, 3.7 cm/s, and 1.85 cm/s. The error bars represent one standard deviation in each direction.

The quasi-steady-state-averaged recoverable sand energy for each trigger height is plotted in Fig. 9, for superficial airflow velocities of 7.4 cm/s, 3.7 cm/s, and 1.85 cm/s. The trend demonstrates that the system at quasi-steady-state yields a roughly constant energy in the extracted sand regardless of front height tested for a given air flow rate. This is attributed to the reactor operational procedure that restricts the volume of fuel smouldered each cycle, approximately 38 mm of fuel height or 7 g of fuel. Thus, the energy produced per mass of fuel is constant and only the reaction rate varies resulting in lower smouldering peak temperatures for slower cycles. The energy extracted does vary with airflow rate, however, indicating that higher airflow rates, which result in higher peak temperatures (see Fig. 5), result in larger amounts of average energy stored in the sand.

There are heat losses from the ejected sand to the ambient air and conical housing, thus energy values are apparatus dependent. The variability between experiments E1, E2, and E3 can be seen in Fig. 9 for a trigger height of 21.2 cm. This may be attributed to slight (random) variability in the fuel feed as any variation in moisture and energy content affects the energy balance of the system. None of the formed fixed carbon is visually observed in the ejected sand for the conditions presented, indicating its total oxidation.

### 3.4 Smouldering performance

#### 3.4.1 The effect of temperature on smouldering velocity

**Fig. 10:**
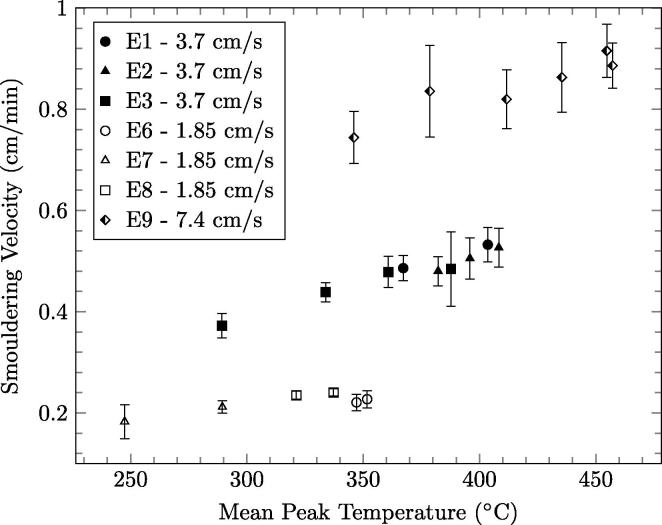
Average smouldering velocity as a function of the near-wall mean peak temperature over quasi-steady-state for 7.4 cm/s, 3.7 cm/s, and 1.85 cm/s. The error bars represent one standard deviation in each direction.

The average smouldering velocity once the reactor reaches quasi-steady-state is plotted as a function of the average peak temperature at quasi-steady-state is shown in Fig. 10 for superficial airflow velocities of 7.4 cm/s, 3.7 cm/s, and 1.85 cm/s. The smouldering velocity is observed to be linearly related to temperature for a given airflow rate, attributed to increased reaction rates for smouldering and pyrolysis. A weaker dependence is observed between temperature and smouldering velocity for a superficial airflow velocity of 1.85 cm/s, illustrated by the difference in slopes presented in Fig. 10, when compared to 7.4 cm/s and 3.7 cm/s. Note that the leftmost symbols in Fig. 10 represent the lowest quasi-steady-state smouldering front heights. Thus, lowering the front will decrease the smouldering velocity due to the lower peak temperatures observed. This matches the observations of the longer cycle times required for lower front heights or for higher fuel moisture content, observed in Fig. 3.

**Fig. 11:**
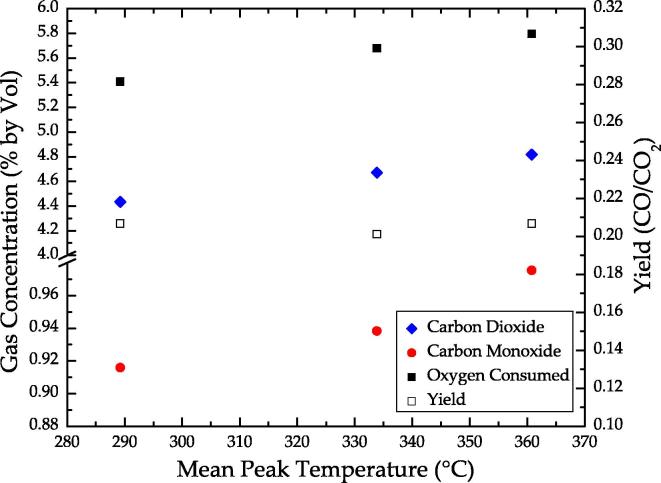
The carbon dioxide and carbon monoxide concentration by volume in the exhaust and the calculated oxygen consumed as a function of mean peak temperature for experiment E3. The right axes show the calculated yield (CO/CO_2_).

The average concentration of carbon dioxide (CO_2_) and carbon monoxide (CO) in the exhaust gases as a function of mean peak temperature for a superficial airflow velocity of 3.7 cm/s is presented in Fig. 11. The oxygen (O_2_) consumed, also presented in Fig. 11, is calculated from the difference between the concentration measured in the exhaust and ambient. For 3.7 cm/s, the carbon dioxide and carbon monoxide concentration range from 4.4% to 4.8% and 0.92% to 0.98% by volume, respectively. Lower reaction rates, as a result of lower smouldering velocity, are characterized by lower oxygen consumption. However, the yield (CO/CO_2_) is observed in Fig. 11 to be independent of the mean peak temperature for the parameters studied; this suggests that the global stoichiometry of the smouldering reactions is relatively independent of mean peak temperature, within the range of operating conditions examined.

#### 3.4.2 The effects of superficial airflow velocity on smouldering

The effect of superficial airflow velocity on smouldering temperature is illustrated in Fig. 5. Higher superficial airflow velocity yields higher peak temperatures, attributed partly to higher oxygen flux to the reaction at higher airflow rates [9], [10], resulting in higher smouldering velocities and higher energy values observed in the extracted sand.

**Fig. 12:**
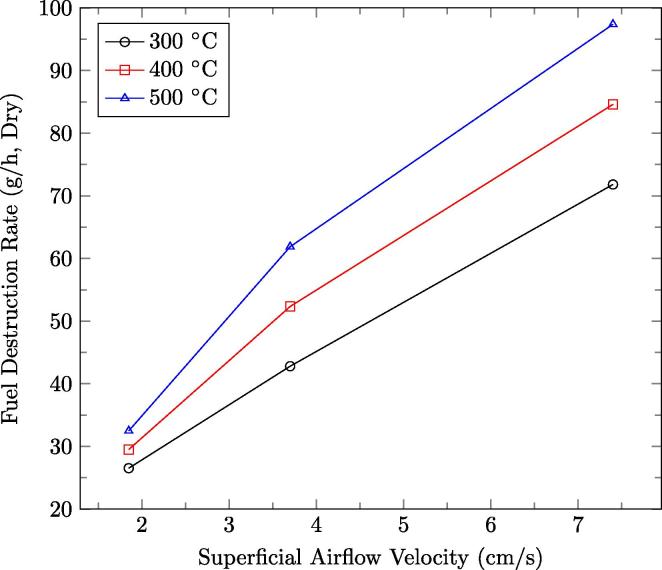
Calculated fuel destruction rate for near-wall peak temperatures of 300 °C, 400 °C and 500 °C as a function of superficial airflow velocity. The linear fit is based on the fuel destruction rate versus temperature dependence measured for 7.4 cm/s, 3.7 cm/s, and 1.85 cm/s.

The effects of superficial airflow velocity on smouldering velocity, which controls the fuel destruction rate, are shown in Fig. 10. The trend shows that the destruction rate, which is proportional to smouldering velocity, is dependent on the mean peak temperature of the smouldering reaction for a given airflow rate. Thus, a linear fit was applied to the fuel destruction rate as a function of temperature for the each of the measured superficial airflow velocities. The destruction rate was then calculated for mean peak (near-wall) temperatures between 300 °C and 500 °C for 7.4 cm/s, 3.7 cm/s, and 1.85 cm/s. The results are presented in Fig. 12. The fuel destruction rate has a stronger dependence on temperature at higher superficial airflow velocities. In this parameter space, the system is observed to be capable of destruction rates from 23.1 g/h to 93.2 g/h for the airflow rates tested. Note that 23.1 g/h is not the lower limit. Fig. 12 underscores that the fuel destruction rate in the continuous reactor can be controlled by adjusting the air flow rate and quasi-steady-state front height, since these control how robust the reaction is (thus the peak temperature and correlated fuel oxidation rate).

A batch process was compared to the continuous process to determine if they share quenching limits at low airflow rates. For this purpose, an additional continuous experiment was conducted with a constant trigger height of 26.3 cm, in which the superficial airflow velocity was consistently lowered over numerous cycles until the reaction quenched. It was possible to run the continuous smouldering experiment with an airflow as low as 0.76 cm/s resulting in a destruction rate and smouldering velocity of 11.9 g/h and 0.11 cm/min, respectively. However, batch experiments consistently fail to ignite at or below a superficial airflow velocity of 1.2 cm/s. The continuous system also, of course, needs to employ a higher superficial airflow velocity than 1.2 cm/s to start-up. But its advantage is that the superficial airflow velocity can be lowered over successive cycles. Extinction usually occurs when the heat generated by the reaction is insufficient to overcome heat losses. One source of heat loss is the reactor walls. This is a fixed loss in a batch process that starts with a cold reactor. However, over a sequence of cycles, the reactor wall temperatures in the continuous reactor approach a quasi steady state, and the major heat loss associated with heating the wall from room temperature is absent. This shifts the energy balance, allowing a lower self-sustaining reaction rate - corresponding to lower air flow rates - in the continuous system.

## 4 Conclusions

The study demonstrates for the first time that continuous smouldering of organic waste in an inert sand matrix is feasible. The system employs intermittent, self-sustaining smouldering reaction in which the fuel oxidation is stopped and then restarted simply by turning the air off and on. Taking advantage of this in a two-phase (forward propagation/downward translation) cycle, essentially indefinite continuous operation of a self-sustaining smouldering reaction was possible. The reactor was shown to operate at long duration (in excess of 16 h and 111 cycles) in continuous operation without extinction, in a self-sustaining manner. The control parameter of injected superficial airflow velocity is shown to be effective at dictating the reaction rates, allowing the operator to adjust the fuel destruction rate.

The robustness of the quasi-steady-state smouldering reaction, as reflected by mean peak temperatures, front velocities, and fuel destruction rates, was observed to increase with (i) distance of the quasi-steady-state front from the base of the reactor, and (ii) increased air flow rate. Quasi-steady-state fuel destruction rates as high as 93 g/h were achieved in this reactor and for these conditions and as low as 12 g/h. The latter was achieved with an airflow rate of 0.11 cm/min, approximately an order of magnitude lower than can support a self-sustaining reaction in an equivalent batch system. The ability to control the fuel destruction rate via airflow manipulation while maintaining self-sustaining smouldering is a critical aspect for implementing this in a sanitation context where the rate of fuel supply is expected to vary. It was demonstrated that excess energy could be recovered from the removed clean sand (up to 140 J/g of sand), and while the amount was not affected by the height of the quasi-steady-state front location, it was maximized by increasing the airflow rate. This provides an opportunity to use that heat for other purposes, further increasing the energy efficiency of the waste treatment system.

The energy balance in smouldering is complex, affected by numerous additional parameters at this scale not studied here including: reactor construction, reactor insulation, boundaries, sand grain size, sand heat capacity, fuel moisture content, fuel organic content, etc. The interaction of these, and the failure points (i.e., where energy losses exceed energy generated) need to be mapped in future work. Also, it is acknowledged that this study uses a surrogate for human feces and future development will involve testing on real feces. However, it is expected that this approach is not only promising for a low cost, low energy toilet to provide sanitation solutions. Rather it may be broadly beneficial for waste management with energy recovery.

## Figures and Tables

**Table 1:** Composition of the surrogate faeces used.

Ingredient	Function or proxy	Energy (kJ/g)	Dry mass (%)
Polyethylene glycol	Water retention	26.1	20
Baker’s yeast	Bacterial debris	18.6	30
Peanut oil	Fat	34.0	20
Miso paste	Protein	16.8	5
Cellulose	Cellulose, fiber	16.7	15
Psyllium husk	Dietary fiber	8	5
Calcium phosphate	Minerals	0	5
Water	Moisture	0	–

**Table 2:** Table of experiments.

Experiment	Airflow rate		Trigger heights studied
	(cm/s)	L/min	cm
E1	3.7	6.8	23.8, 21.2
E2	3.7	6.8	26.3, 23.8, 21.2
E3	3.7	6.8	21.2, 18.7, 16.2, 13.6
E4	3.7	6.8	26.3
E5	3.7	6.8	13.6
E6	1.85	3.4	26.3, 23.8
E7	1.85	3.4	21.2, 18.7
E8	1.85	3.4	16.2, 13.6
E9	7.4	13.6	26.3, 23.8, 21.2, 18.7, 16.2, 13.6
E10	3.7	6.8	Batch
